# A survey of suicide literacy in Japanese school teachers

**DOI:** 10.1038/s41598-023-50339-2

**Published:** 2023-12-27

**Authors:** Satoshi Yamaguchi, Jerome Clifford Foo, Tsukasa Sasaki

**Affiliations:** 1 Department of Physical and Health Education, Graduate School of Education, The University of Tokyo, Tokyo, Japan; 2 Unit for Mental Health Promotion, Research Center for Social Science & Medicine, Tokyo Metropolitan Institute of Medical Science, Tokyo, Japan; 3 Institute for Psychopharmacology, Medical Faculty Mannheim, Central Institute of Mental Health, University of Heidelberg, Mannheim, Germany; 4 Department of Genetic Epidemiology in Psychiatry, Medical Faculty Mannheim, Central Institute of Mental Health, University of Heidelberg, Mannheim, Germany; 5 Department of Psychiatry, College of Health Sciences, University of Alberta, Edmonton, Canada

**Keywords:** Health care, Risk factors, Signs and symptoms

## Abstract

School teachers are in a unique position to recognize suicide-related problems in their students and to appropriately support them; teachers may need high levels of suicide literacy. However, few studies have examined current levels of suicide literacy in teachers. This study aimed to investigate suicide literacy in school teachers. Teachers (n = 857) from 48 Japanese schools (primary and junior-/senior-high) answered a self-administered questionnaire assessing (a) knowledge about suicide, (b) intention to ask about students’ suicidal thoughts/plans, and (c) attitudes towards talking to students with mental health problems. The average proportion of correct answers to the knowledge questions (10 items) was 55.2%. Over half of the teachers knew that suicide is a leading cause of death in adolescents (55.0%), and that asking about suicidality is needed (56.2%). Half of the teachers intended to ask students about their suicidal thoughts (50.2%) and fewer intended to ask about experiences of planning suicide (38.8%). Most of the teachers (90.4%) agreed with the idea that talking to students with mental health problems was a teacher’s responsibility. Intention to ask about students’ suicidal thoughts/plans were higher in teachers in their 20s (vs. 40s–60s) and working at junior-/senior-high schools (vs. primary schools). Suicide literacy in Japanese school teachers was observed to be limited. However, teachers felt responsibility for helping students with mental health problems. The development and implementation of education programs may help improve teachers’ suicide literacy, which, in turn, could encourage effective helping behaviors of teachers for students struggling with suicidality.

## Introduction

Suicide is a leading cause of death in adolescents^1^ and can be closely linked to mental health problems^2–4^, which start to sharply increase in prevalence during this important and vulnerable period^5^. However, the majority of adolescents are likely to be reluctant to disclose their intention to die by suicide^3,4^ and to seek help for their mental health problems^6^. Considering that adolescents spend a majority of their time in schools, school teachers are expected to play a gatekeeping role for adolescent suicide, that is, recognizing adolescents’ mental health problems including suicidality, and supporting help-seeking behaviors^7,8^. To play this role, teachers need to have sufficient knowledge about and more understanding attitudes towards suicide and mental health problems/illnesses.

Knowledge about and attitudes towards mental health/illnesses are defined as mental health literacy (MHL)^9^, and this definition of MHL has been adapted to suicide (hereafter, suicide literacy)^10^. MHL comprises several components, including the ability to recognize specific disorders, knowledge of risk factors and causes, and having attitudes that promote recognition and appropriate help-seeking^9,11^. Based on these components of MHL, the following components of suicide literacy have been evaluated: knowledge (e.g., warning signs, risk factors, prevention): confidence (e.g., in asking about suicidality, in providing help): and attitudes (e.g., beliefs in whether suicide is preventable, and in whether suicide risk should be directly asked about)^7^. Teachers with high suicide literacy as well as MHL may be able to appropriately recognize warning signs of suicide and mental health problems in students, which may enable these students to receive early and appropriate care^12–14^, although the relationship between knowledge about suicide and how it translates to behavioral change remains unclear^7^.

Thus far, a number of studies have examined MHL in teachers^15–29^; these studies have observed that the majority of teachers had limited knowledge about^15,22,23,26,28^ and recognition of^15,17,25,27^ mental illnesses, and low confidence^15,21^ in helping students with mental health problems. Several studies have examined suicide literacy in teachers^30–37^; these studies have also observed that the majority of teachers had limited knowledge about suicide^30–34,36,37^ and low confidence in helping students with suicidality^35^. However, most of the studies about suicide literacy in teachers were conducted nearly/over 15 years ago^30–34^, conducted primarily in North America^32,33,35^ and Australia^30,31,34^, and/or did not investigate youth-relevant suicide literacy but suicide literacy in general^30,32,36,37^. Also, these studies only reported aggregate scores of overall/subcategories (e.g., risk factors, warning signs) of suicide literacy without indicating whether the items, which covered diverse topics (e.g., risk factors/warning signs consisted of mental illnesses, lack of social support, and past experience of suicidal attempts), should be summarized into a single construct (e.g., by factor analysis)^30–32,37^. Importantly, without detailed information about the questions and the responses to them, the specific knowledge that the teachers lacked and which need to be taught are not known. According to a recent systematic review of suicide gatekeeper training programs^7^, there have been few programs designed specifically for teachers (i.e., the majority of the programs were developed for suicide prevention in general, such as the Question, Persuade, Refer program^38^). Future suicide literacy programs for teachers need to be designed with the awareness of youth-specific risks and also to equip teachers with the knowledge and skills to deliver interventions that are effective in youth^7^. Research efforts need to identify the most important points of knowledge that are effective in causing the desired changes in suicide literacy/teacher thinking and behavior, and optimize the delivery of that knowledge. To develop effective programs, assessing current levels of specific knowledge about adolescent suicide among teachers is a reasonable first step, and further research in more diverse populations and communities worldwide is needed.

In Japan, suicide is the leading cause of death in adolescents^39^, and the suicide rate increased from 3.8 per 100,000 in 1990 to 9.9 per 100,000 in 2019 for 15–19 year olds^40^, much higher than average and median global suicide rates (6.1 and 4.2 per 100,000 in 2019, respectively^41^), a situation which requires more systemic attention. Japan does not have a system of general practitioners^42^ and there are few full-time school counselors/psychologists^43^ or other easy-to-reach professionals from whom adolescents may access mental health services (as available in other countries^44^). In this situation, teachers in Japan have the potential to play important roles in helping students with suicidality and other mental health problems; improving teacher suicide literacy and MHL may be an important step in empowering them to help at-risk adolescents.

Understanding the relationship between the different components of suicide literacy is also important. Previous studies have only examined associations between “knowledge” and “attitudes” (stigma towards people with suicidality)^45–47^. Considering that the “intention” (included in the “attitudes” component) to do something predicts actual future behaviors^14,48^, an important next step is to investigate the relationship between “knowledge” and the intention to directly ask about suicide risk; asking can be crucial in suicide prevention^3,4,38,49–51^. One open question is whether having more knowledge and awareness leads to higher intention to ask, or whether having the intention to inquire about suicide reflects more motivation to prevent it, leading an individual to seek out knowledge.

The aim of the current study is to assess current levels of specific knowledge about adolescent suicide and intention to ask about students’ suicidal thoughts/plans in Japanese teachers. These knowledge and intention are needed to develop effective gatekeeper training programs for teachers to recognize suicidality in their students and appropriately support them. Additionally, we examined associations between knowledge about adolescent suicide and intention to ask about students’ suicidal thoughts/plans while also examining the effects of demographic variables (e.g., age and sex) which have been observed to affect suicide literacy in adult samples (not limited to teachers)^45–47,52–54^.

## Methods

### Procedure and participants

In 2020, the Board of Education of Saitama prefecture (population: 7 million) informed all public schools in their jurisdiction about the current study. The principals of 48 prefectural or municipal schools (20 primary schools, 18 junior high schools, and 10 senior high schools) told the Board that they wanted their schools to participate in the study; in these schools, 58.6% (n = 857) of teachers participated in the current study.

### Contents of the questionnaire

Suicide literacy in teachers was assessed using a self-administered questionnaire. The questionnaire was drafted by one of the authors (TS) and edited and refined by a team of psychiatrists, psychologists (including JCF), teachers (including SY) and school nurses. The questionnaire was written in Japanese, and comprised the following 4 parts.

#### Part 1: Demographic variables

Demographic information of teachers was assessed. The information included age, sex, school type (primary school, junior high school, or senior high school), academic degree, previous participation in mental health seminars, and experience of dealing with someone suffering from a mental illness (Table 1).

**Table 1:** Demographic data of school teachers.

Characteristic	Number (%)
Total	857
Age	
20s	256 (29.9)
30s	234 (27.3)
40s	119 (13.9)
50s	156 (18.2)
60s	91 (10.6)
No answer	1 (0.1)
Sex	
Male	483 (56.4)
Female	373 (43.5)
No answer	1 (0.1)
School type	
Primary school	224 (26.1)
Junior high school	344 (40.1)
Senior high school	287 (33.5)
No answer	2 (0.2)
Academic degree	
Associate degree^a^	66 (7.7)
Bachelor	711 (83.0)
Masters (or higher)	75 (8.8)
No answer	5 (0.6)
Previous participation in mental health seminars	
None	683 (79.7)
Once or more times	173 (20.2)
No answer	1 (0.1)
Experience of dealing with someone suffering from a mental illness	
No	305 (35.6)
Yes	510 (59.5)
No answer	42 (4.9)

^a^An associate degree is an undergraduate degree in Japan awarded after a course of post-secondary study lasting 2 or 3 years.

#### Part 2: Knowledge about suicide

The second part of the questionnaire comprised 10 questions regarding knowledge about suicide (Table 2), including basic knowledge about the epidemiology, risk factors and care/treatment of suicide, based on vital statistics in Japan^39^ and previous studies^2–4,7,49–51,55,56^. The possible answers to these questions were: “True”, “False”, or “I don’t know”. Correct answers were scored 1 (otherwise scored 0) and the scores were added up. In the present sample, the internal consistency (Cronbach’s alpha) of the questions was 0.63.

**Table 2:** School teachers’ knowledge about suicide.

Items	Correct answer	Proportion of correct responses (%)
Asking about suicidal ideation should be avoided, because it can lead to suicide attempts	F	56.2
In Japan, suicide is the first leading cause of death in older teens	T	55.0
Paying attention to students who repeatedly self-harm is the most important in suicide prevention	F	56.1
Asking about suicidal thoughts should be left to experts, and teachers should not ask them	F	71.1
Risk of suicide does not differ depending on whether people have someone to discuss their worries with or not	F	80.0
For suicide prevention, the same amount of attention should be paid to a person whether or not they have a history of suicide attempts	F	15.4
If someone told you about a suicidal plan and were begged not to tell it to anyone, you should not tell the plan to anyone else	F	84.3
The focus of suicide prevention efforts should be primarily on students being treated by mental health specialists including psychiatrists	F	58.0
When teachers examine a student’s risk of suicide, they should not ask whether there is a specific suicidal plan,	F	37.6
When someone has a very high risk of suicide, the person should be hospitalized, even if they do not agree	T	40.4
Average percentage of correct answers to the questions about suicide (SD)		55.2 (21.9)

*F* false, *T* true, *SD* standard deviation.

#### Part3: Intention to ask about students’ suicidal thoughts/plans

Teachers were asked to read a case vignette describing a teenage student (Student A) with suicidal thoughts. The vignette was adapted from Jorm et al.^12^.

The description of the vignette is as follows. “Student A feels that they will never be happy again and believes that their family would be better off without them. They have had feelings of hopelessness and have constantly been thinking of ways to end their life. They have also been up to a rooftop with the intention to jump.”

Having read this vignette, teachers were asked to what extent they agreed with the 5 items (see Table 3) regarding intention to ask students who were in a similar situation to student A about their suicidal thoughts/plans. There were 4 answer choices, and these choices were scored as follows: “Strongly agree” (4), “Agree” (3), “Disagree” (2), “Strongly disagree” (1). Higher score indicated higher intention. The total score of the 5 items was used for statistical analyses. In the present sample, the internal consistency (Cronbach’s alpha) of the items was 0.92.

**Table 3:** School teachers’ intention of asking students about their suicidal thoughts and plans.

Items	Proportions of responses (%)			
	Strongly agree	Agree	Disagree	Strongly disagree
Ask whether a student does not want to live	17.4	35.5	23.3	23.8
Ask whether a student wants to die	15.7	34.5	24.2	25.6
Ask whether a student has thought about how to die by suicide	12.6	26.2	33.2	28.0
Ask whether a student has prepared to die by suicide	12.6	29.7	31.2	26.5
Ask whether a student has actually done something or been somewhere with the intent to die	14.3	33.8	28.2	23.7

‘A student’ in items above refers to one in a similar situation to Student A in the vignette.

#### Part 4: Attitudes towards talking to students with mental health problems

In the fourth part, teachers were asked the following question: “How confident do you feel in talking to students with mental health problems?” Possible answers to this question were “Fully confident”, “Confident”, “Not very confident”, “Not confident at all”. The teacher was considered to have confidence, when the answer was “Fully confident” or “Confident”. In addition, teachers were asked “What extent do you agree with the idea that talking to students with mental health problems is teachers’ responsibility?” Possible answers to the question were “Strongly agree”, “Agree”, “Disagree” or “Strongly disagree”. The teacher was considered to agree with the idea when the answer was “Strongly agree” or “Agree”.

### Statistical analysis

Demographic statistics were compiled and responses to the questionnaires were evaluated. Multilevel regression analyses were conducted to examine whether knowledge about suicide (*knowledge*) had an effect on intention to ask about students’ suicidal thoughts/plans (*intention*) in teachers, or vice versa, while also examining effects of demographic variables. All demographic variables shown in Table 1 were included in the models. A random effect of intercept for school was included in the analyses, since the teachers were sampled from 48 different schools. The level of significance was set at alpha = 0.05. The analyses were performed using R version 4.1.3 with the lme4 and lmerTest package.

### Ethics approval and consent to participate

The aim and contents of the study were explained to participating teachers in writing, and we obtained written informed consent from all the teachers who participated. The study was approved by The University of Tokyo Human Research Ethics Committee (#18–48). This study was conducted in accordance with the Declaration of Helsinki.

## Results

### Demographic variables

Table 1 shows demographic data of participating teachers. More than half of the teachers were in their 20s or 30s (57.2%) and male (56.4%). Completion of a Bachelor’s degree was the highest education level for most of the teachers (83.0%). One fifth of the teachers (20.2%) previously participated in a mental health seminar once or more. More than half of the teachers previously had experiences of dealing with someone suffering from a mental illness (59.5%).

### Knowledge about suicide

Table 2 shows the proportion of correct answers to the knowledge questions about suicide. The average proportion of correct answers was 55.2% (standard deviation = 21.9%). Regarding specific questions, seven out of ten items had low proportions (around or below half) of correct answers: for example, the fact that suicide is the leading cause of death among older teens (aged 15–19) in Japan (55.0%); the need to ask about suicidal ideation (56.2%) and specific suicide plans (37.6%); the fact that a previous suicide attempt is a significant risk factor for suicide (15.4%); that the focus of suicide prevention efforts does not have to be primarily on students who repeatedly self-harm (56.1%), and who are currently receiving treatment by mental health specialists (58.0%).

### Intention to ask about students’ suicidal thoughts/plans

Table 3 shows intention of the teachers to ask students who are similar to Student A in the vignette about their suicidal thoughts/plans. Regarding suicidal thoughts, half of the teachers disagreed or strongly disagreed to ask students if they did not wish to live (47.1%) or wished to die (49.8%). Regarding suicidal plans, a majority of teachers disagreed or strongly disagreed to ask students about their experiences of thinking about how to die by suicide (61.2%) and of preparing for suicide (57.7%).

### Attitudes towards talking to students with mental health problems

Most of the teachers (90.4%) agreed with the idea that talking to students with mental health problems was a teachers’ responsibility. However, less than half of the teachers (43.6%) answered that they had the confidence in talking to students with such problems.

### Association between knowledge about suicide and intention to ask about students’ suicidal thoughts/plans

Table 4 shows the results of the multilevel regression analyses examining whether *knowledge* had an effect on *intention* in teachers, or vice versa, in addition to examining the effects of demographic variables on *knowledge* and *intention*. Teachers with higher levels of *intention* had significantly higher *knowledge* (unstandardized coefficient (B) = 0.15, 95% confidence interval (CI): 0.11–0.18), while no demographic variables had significant effects on *knowledge*. In the analysis where *intention* was modeled as the outcome, it was observed that teachers with higher *knowledge* had significantly higher levels of *intention* (B = 0.57, 95% CI 0.44–0.71). Also, teachers in their 40s (B = − 1.02, 95% CI − 1.98 to − 0.05), 50s (B = − 1.98, 95% CI − 2.88 to − 1.08) and 60s (B = − 1.58, 95% CI − 2.64 to − 0.52) had lower *intention* compared to teachers in 20s. *Intention* was higher among teachers working at junior-high schools (B = 0.79, 95% CI 0.02–1.55) and senior-high schools (B = 1.20, 95% CI 0.36–2.03) compared to teachers working at primary schools. There were no significant effects of sex, previous experiences of participating mental health seminar, and previous experience of dealing with someone suffering from a mental illness on *intention*.

**Table 4:** Differences in knowledge about suicide and intention to ask about students’ suicidal thoughts/plans among teachers by demographic data.

Variable	Knowledge about suicide (Score range: 0–10)	Intention to ask about suicidal thoughts/plans (Score range: 5–20)^a^
	Unstandardized coefficients (95% confidence interval)	
Components of suicide literacy		
Knowledge about suicide	–	0.57*** (0.44, 0.71)
Intention to ask about suicidal thoughts/plans	0.15*** (0.11, 0.18)	–
Demographic variables		
Age (reference = 20s)		
30s	0.06 (− 0.34, 0.46)	− 0.47 (− 1.26, 0.32)
40s	0.08 (− 0.42, 0.57)	− 1.02* (− **1.98, **− **0.05)**
50s	0.12 (− 0.34, 0.59)	− 1.98*** (− **2.88, **− **1.08)**
60s	− 0.03 (− 0.57, 0.51)	− 1.58** (− **2.64, **− **0.52)**
Female (reference = male)	− 0.10 (− 0.42, 0.22)	0.14 (− 0.49, 0.77)
School type (reference = primary school)		
Junior high school	0.24 (− 0.15, 0.63)	0.79* (0.02, 1.55)
Senior high school	− 0.10 (− 0.53, 0.33)	1.20** (0.36, 2.03)
Academic degree (reference = bachelor)		
Associate degree^b^	− 0.55 (− 1.14, 0.03)	0.49 (− 0.66, 1.64)
Master’s degree	0.52 (− 0.02, 1.06)	− 0.24 (− 1.31, 0.82)
Previous participation in mental health seminars	0.01 (− 0.39, 0.41)	− 0.14 (− 0.92, 0.64)
Any experience of dealing with someone suffering from a mental illness	0.16 (− 0.16, 0.48)	0.34 (− 0.29, 0.97)

*p < 0.05; **p < 0.01; ***p < 0.001.

p values were derived from multilevel linear regression analyses including all variables at the same time.

^a^Higher score indicates higher intention.

^b^An undergraduate degree in Japan awarded after a course of post-secondary study lasting 2 or 3 years.

## Discussion

The current study investigated suicide literacy in Japanese school teachers. The level of the literacy in Japanese teachers may be limited; proportions of the correct answers were around 50% or lower to most of the suicide knowledge questions, and around half of the teachers would not ask students with suicidality about their suicidal thoughts/plans. On the other hand, most of the teachers regarded talking to students with mental health problems as a part of their responsibility, although their confidence in talking about such problems with the students is low. Considered together, improving suicide literacy in teachers may be a first step for them to actually help students with suicidality.

Regarding the specific knowledge, approximately half of the teachers did not know the fact that suicide is the leading cause of death among older teens (aged 15–19) in Japan^39^, which may suggest teachers’ low awareness of or familiarity with suicide-related problems in adolescents. Considering that one of major risk factors is mental health problems^2–4^, which are prevalent during adolescence^5^, a large number of students are potentially at risk of suicide. A recent meta-analysis reported that the 12-month prevalence of suicidal ideation was 14.2%, and that of suicide attempts was 4.5% in adolescents worldwide^57^. Knowledge of these facts may help teachers perceive suicide-related problems as personally relevant, which, in turn, may lead to them engaging in preventive behaviors^58^. This knowledge could be provided to teachers through suicide literacy training programs.

Also, approximately half of the teachers thought that asking about suicidal ideation could lead to a suicide attempt. These types of effects have not been seen in previous studies^49–51^. Asking students about the ideation may be needed for teachers to proactively recognize and intervene in cases of student suicidality, since the majority (71–76%) of those who die by suicide tend not to disclose their intention to die by suicide^3,4^.

Furthermore, around half of the teachers did not know that the focus of suicide prevention should also be on students who do not clearly show mental health related problems (i.e., repeating self-harm, receiving treatment by mental health specialists); although self-harm and mental illnesses are risk factors of suicide^2–4^, the majority (79%) of those who die by suicide appear not to experience self-harm^4^ and are not (70–72%) receiving treatments for mental illnesses^3,4^. Focusing only on these factors may lead to overlooking many students with suicide risk. Teachers need to know these facts, and need to talk to students about any concerns such as mental health problems when students seem to have these concerns. Teachers equipped with such knowledge may be willing to talk to students about their concerns, considering that most of the teachers felt responsibility for helping students with mental health problems.

Around half of the teachers indicated that they would not ask students with suicidality about their suicidal thoughts/plans. This number is concerning, considering that asking about suicide is a crucial part of suicide prevention^3,4,38,49–51^. Increasing this intent may be important to encourage teachers to take action, as attitudes (including the intention of doing something) predict future actual behaviors^14,48^. In our models, we observed that *knowledge* had a significant effect on *intention*, and *intention* also had a significant effect on *knowledge*. It may be possible both that providing teachers with knowledge about adolescent suicide may lead to increased intention to ask about students’ suicidal thoughts/plans, and that improving *intention* may motivate teachers, leading to investing more effort to gain *knowledge*. Due to the cross-sectional nature of this design, we cannot evaluate the directionality of these effects. Still, our study provides preliminary evidence about the associations between *knowledge* and *intention*, and suggests that bidirectional effects exist; *knowledge* and *intention* are likely to be linked and both important components of literacy. Future research will need to look closer at this relationship.

Regarding the effects of demographic variables, junior- and senior-high school teachers expressed higher levels of *intention* compared to primary school teachers. In Japan, the age of students in junior- and senior-high schools ranges from 12–18, where the number of suicide death sharply increases^39^; suicide related issues may draw more attention in teachers in these schools compared to teachers in primary schools. Teachers’ age, sex, and education level had no significant effects on their *knowledge*. None of these factors were observed to have significant effects in one previous study investigating the general population^53^, but other studies have observed that all^52^ or some^45–47^ of these factors had significant effects; younger age^45–47,52^, female sex^52^, and higher education level^46,47,52^ had positive effects on *knowledge*. Further studies are needed to clearly understand the effects of these factors. Regarding education level, a limited range in teachers (most teachers had Bachelor’s degrees) may have resulted in its non-significant effect; previous studies observing a significant effect of education level in the general population included more varied education levels (e.g., high school or less)^46,47,52^. Previous participation in mental health seminar did not have a significant effect on *knowledge* or *intention*. Thus far, a number of MHL programs for teachers have been developed^59^; however, only a some of the programs address suicide^60–66^, and few suicide prevention trainings have been developed specifically for teachers^7^. More suicide literacy programs for teachers need to be developed and implemented.

### Limitations

The current study has several limitations. First, participants were school teachers from a single prefecture in Japan. Caution may be needed when generalizing these results to other populations. Second, the participation rate was not high (58.6%), although the rate is not lower than our previous study (53.3%) investigating MHL in high school teachers from another prefecture^15^. Suicide literacy in teachers who decided to participate might differ from the literacy in those who did not. Third, the questionnaire used in the current study was newly developed and tailored to assess suicide literacy in teachers through discussion with specialists in mental health and adolescents such as psychiatrists, psychologists, teachers and school nurses.

## Conclusions

Suicide literacy in the Japanese school teachers (from primary, junior-/senior-high schools) may be limited; they had insufficient knowledge about adolescent suicide and low intention to ask students with suicidality about their suicidal thoughts/plans. However, most of the teachers regarded talking to their students with mental health problems as a part of their responsibility, suggesting that they may be willing to act if properly prepared. Programs which effectively provide teachers with suicide literacy may help teachers notice students with suicidality and appropriately support them. Efforts should be made to incorporate these suicide literacy programs in teacher training, not only for in-service teachers but also for pre-service teachers at the university level. To do so, help will be needed at the level of educational policy and governance, by urging educational boards and ministries to take action.

## Data Availability

The datasets generated and/or analyzed during the current study are not publicly available due to data protection and privacy regulations of the Saitama Prefectural Board of Education. They may be made available from the corresponding author on reasonable request.

## Abbreviation

MHL: Mental health literacy
